# Nurses’ and managers’ perceptions of quality nursing care in public hospitals in Ghana

**DOI:** 10.4102/hsag.v31i0.3381

**Published:** 2026-05-30

**Authors:** Ba-Etilayoo Atinga, Wilma ten Ham-Baloyi, Nolundi Radana

**Affiliations:** 1 Department of Nursing, University of Energy and Natural Resources, Sunyani, Ghana; 2 Department of Nursing Science, Faculty of Health Sciences, Nelson Mandela University, Gqeberha, South Africa

**Keywords:** Ghana, nurse managers, nurses, perceptions, quality nursing care

## Abstract

**Background:**

Quality improvement is a global healthcare priority. In Ghana, consistent delivery of high-quality nursing care remains challenging, and the perspectives of nurses and nurse managers are often underrepresented in discussions on care quality. Exploring these perspectives is essential to inform practice, management and policy.

**Aim:**

This study explored nurses’ and nurse managers’ perceptions of quality nursing care in public hospitals in the Bono Region of Ghana.

**Setting:**

The study was conducted at three public hospitals in Ghana.

**Methods:**

A qualitative, exploratory–descriptive design was adopted. Semi-structured face-to-face interviews were conducted with 18 professional nurses and three nurse managers purposively selected from three public hospitals. Data were analysed thematically using Tesch’s method, guided by Creswell’s qualitative data analysis framework.

**Results:**

Three themes emerged: (1) attributes of quality nursing care, including individualised, ethical and evidence-based practice; (2) barriers to quality care, such as inadequate resources, limited supervision and negative staff attitudes; and (3) the impact of nursing care on patient outcomes and healthcare delivery. Interpersonal relationships and organisational constraints were found to influence care quality.

**Conclusion:**

Quality nursing care is underpinned by clinical competence, ethical practice, teamwork and institutional support. Gaps in these areas compromise patient outcomes and staff satisfaction. Strengthening professional development, ethical standards, collaboration and organisational support is essential to enhance care quality.

**Contribution:**

This study provides context-specific insights into quality nursing care from the perspectives of nurses and nurse managers in Ghana, contributing evidence to inform nursing management practices, policy development and quality improvement initiatives in similar resource-constrained settings.

## Introduction

Quality improvement in healthcare has become a global imperative, with growing emphasis on integrating the perspectives of both patients and care providers in evaluating and delivering care (Ostern et al. 2021). Despite this global momentum, many low- and middle-income countries (LMICs), including Ghana, continue to face challenges in consistently delivering high-quality nursing care that meets patients’ expectations (Danso et al. 2024). Across settings, patients expect compassionate, competent and timely care from nurses – professionals who remain central to achieving positive health outcomes and patient satisfaction (Lee & Seo 2022).

The Institute of Medicine (IOM 2013) defines quality care as care that increases the likelihood of desired health outcomes and is consistent with current professional knowledge. This perspective aligns with the argument by Lachman, Batalden and Vanhaecht (2020) that quality must reflect the values and expectations of both patients and healthcare providers. Quality nursing care is therefore grounded in professional competence, ethical conduct, attentiveness and the ability to deliver care that is safe, timely, efficient, equitable and person-centred (Stavropoulou et al. 2022).

Nurses are foundational to healthcare systems, serving as frontline providers responsible for patient assessment, timely intervention, health education and emotional support. Their capacity to deliver safe and effective care is especially critical in resource-constrained settings. Studies indicate that nurses often employ adaptive strategies, such as teamwork and task prioritisation, to maintain care quality under pressure (Al-Akash et al. 2024). Nurse managers further strengthen these efforts by translating administrative policies into clinical practice, facilitating staff training and leading quality improvement initiatives (Agbanu 2023). Their leadership in areas such as resource allocation, performance management and staff support are essential for sustaining staff motivation and improving service delivery outcomes (Acheampong & Domfeh 2021).

In Ghana, public hospitals serve as the main access points for healthcare, providing services to most of the population through government subsidies and the National Health Insurance Scheme (Sarfo 2020). The Ghana Health Service’s Quality Assurance Programme exemplifies national efforts to improve care standards by involving both nurses and nurse managers in policy implementation and service evaluation (Kumah 2024). While nurses focus primarily on direct patient care – including treatment administration, patient monitoring, ethical practice and family education – nurse managers are responsible for supervision, resource allocation, staff development and ensuring compliance with clinical protocols.

Despite these initiatives, concerns about declining nursing care standards persist, particularly in the study setting.

Patients have reported feeling unsafe during hospitalisation, especially during weekends when staff shortages and lapses in care are more common (Konlan et al. 2020). Ongoing challenges such as neglect, medication errors, inadequate infection control and ineffective communication continue to undermine patient safety and public trust.

These systemic issues highlight the need for targeted, context-specific interventions to improve nursing care delivery.

Although patient perspectives on quality nursing care in LMICs are well documented (Afulani et al. 2023; Kassa et al. 2022; Makua, Mokoena & Mkhize 2024; Ojebode, Akin-Otiko & Sobo 2021), the voices of nurses and nurse managers – the primary providers of care – remain underrepresented in quality improvement discourse. Abugre and Bhengu (2024) report that nurse managers view patient-centred care as central to improving both care quality and nurse job satisfaction.

Conversely, Ansah Ofei and Paarima (2021) demonstrate how ineffective leadership practices negatively affect staff morale and performance, while Kumah (2024) highlights the influence of management approaches on nurse retention and professional commitment. These findings underscore the critical role of supportive leadership in shaping work environments and care outcomes.

Understanding the perspectives of nurses and nurse managers is therefore essential for identifying systemic barriers – such as resource limitations, communication gaps and inadequate supervision – that hinder effective care delivery. Insights from these groups can inform the development of contextually appropriate strategies to address such challenges, strengthen professional development and enhance healthcare system performance. This study explores how nurses and nurse managers perceive quality nursing care in public hospitals in Ghana’s Bono Region, with the aim of generating evidence-based recommendations to improve nursing practice and healthcare delivery in similar LMIC contexts.

## Research methods and design

### Research design

A qualitative, descriptive-exploratory design was employed to explore nurses’ and nurse managers’ perceptions of the quality of nursing care in public hospitals within the Bono Region of Ghana. This design enabled an in-depth understanding of participants’ experiences, perspectives and motivations regarding care delivery.

### Theoretical framework

The study was underpinned by the Donabedian model of quality care (Donabedian 1988), which informed the development of the interview guide, thematic organisation and interpretation of findings. This model conceptualises quality across three interrelated dimensions: structure (e.g. resources, staffing, infrastructure), process (e.g. clinical practices, interactions) and outcomes (e.g. patient satisfaction, recovery). It provided a systematic lens to examine how healthcare systems and personnel influence care delivery.

### Study setting

The study was conducted in three purposively selected public hospitals – two district hospitals and one regional hospital – within a municipality of the Bono Region, Ghana. These hospitals were chosen based on their provision of comprehensive services (e.g. internal medicine, paediatrics, obstetrics, orthopaedics) and their central role in public healthcare delivery in the region.

### Participants and sampling

Nurses and nurse managers were selected purposively based on specific eligibility criteria. Nurses were eligible to participate if they were employed at one of the selected hospitals and actively engaged in patient care. Nurse managers were included if they held supervisory roles and were part of hospital leadership. Participants were required to speak English fluently and have at least 3 years of professional experience.

### Recruitment

Recruitment targeted professional nurses directly involved in patient care within medical and surgical wards, and nurse managers (typically matrons) responsible for supervising nursing staff. Researchers and trained assistants approached potential participants in staff tea rooms or offices to explain the study and share participant information sheets. Interested individuals were scheduled for interviews at times and locations of their choice. Written informed consent was obtained on the day of the interviews.

### Data collection

Data were collected from March 2023 to April 2023 through 21 face-to-face semi-structured interviews – 18 with professional nurses and three with nurse managers. The interview guide, underpinned by the Donabedian model of quality care dimensions (Donabedian 1988), was developed based on relevant literature and reviewed by two senior researchers. The interview guide included open-ended questions exploring perceptions, barriers, facilitators and outcomes of quality nursing care. Probing and paraphrasing were carried out to elicit in-depth information. The guide was piloted with two nurses and one nurse manager, and because no revisions were necessary, the pilot data were included in the final analysis. Interviews were conducted in English by the first author, a male researcher independent of the study participants and experienced in qualitative interviewing. The researcher was supported by a trained, independent field researcher with expertise in qualitative research to take fieldnotes. Each session, lasting between 40 min and 60 min, was held in a private hospital office without bystanders to ensure confidentiality. No follow-up interviews needed to be conducted. Data collection proceeded until thematic saturation was achieved, at which point no additional themes were identified. This point was reached after the 17th interview with professional nurses and the 2nd interview with nurse managers. To confirm the findings, one additional interview was conducted with each group.

### Data analysis

Within 1 week of each interview, a professional transcriber produced verbatim transcripts of the audio recordings. The second and third authors conducted quality checks of the transcripts. Transcripts were integrated with field notes and analysed using Creswell’s thematic analysis approach, which involved organising data, reading and re-reading transcripts, coding relevant segments, and identifying emergent categories and themes (Creswell & Poth 2018). The first author and an independent coder conducted the analysis. Each participant was assigned a unique identifier that included details such as facility, age, gender, rank and years of experience.

### Trustworthiness

To ensure rigour and minimise bias, the authors employed several methodological strategies, including memoing, peer debriefing and consultation with qualitative research experts external to the study team. In addition, this study was guided by Lincoln and Guba’s framework of trustworthiness (Polit & Beck 2021), which encompasses credibility, dependability, confirmability, transferability and authenticity.

To enhance analytical consistency and reliability, all transcripts were independently coded by the second researcher. Member checking was conducted with three participants to verify the accuracy of transcripts and confirm the credibility of emerging themes. Dependability and confirmability were further supported through the maintenance of a comprehensive audit trail, which included field notes, reflexive memos and documentation of coding decisions.

To facilitate transferability, the authors provided rich, contextualised descriptions of participants’ experiences, enabling readers to assess the applicability of the findings to similar settings. Authenticity was maintained by ensuring a balanced representation of diverse perspectives across different facility types and professional roles.

### Ethical considerations

Ethical clearance to conduct this study was obtained from the Nelson Mandela University Research Ethics Committee on 12 September 2022. The ethical clearance number is H22-HEA-NUR-008. Ethical clearance was also received from the Kintampo Human Research Committee in Ghana on 08 February 2023. The ethical clearance number is KHRCIEC-2023-02. Written permissions were obtained from hospital administrators. Confidentiality was ensured by conducting interviews in private settings, anonymising data using pseudonyms and securing confidentiality agreements with the transcriber and fieldworker. Informed consent was obtained from all participants before data collection.

## Results

### Participants’ profile

A total of 21 participants were interviewed, comprising 18 professional nurses and three nurse managers from three public hospitals in the Bono Region of Ghana. The participants’ profile for both groups of participants is described next.

#### Professional nurses

A total of 18 professional nurses were included (six from each hospital). Participants’ ages ranged from 27 to 40 years, with the majority being female (66.7%, *n* = 12). Most participants were married (83.3%), while a smaller proportion of participants were single. In terms of educational qualifications, half of the nurses held Bachelor’s degrees, while the remainder had diplomas in nursing. All participants were registered general nurses, with a few having additional specialisations in paediatrics and women’s health. Their professional ranks ranged from staff nurse to principal nursing officer, reflecting a mix of junior and senior positions. Years of experience varied from 3 to 12 years, with most participants having more than 6 years of professional experience.

#### Nurse managers

The nurse managers comprised three participants (one from each hospital). All were female, married and aged between 52 and 57 years. They were highly experienced, each having over 20 years of professional practice.

All participants held the position of Deputy Director of Nursing Services, reflecting senior leadership roles. Their educational qualifications ranged from Bachelor’s to Master’s degrees, and all possessed advanced professional certifications, including midwifery, public health nursing and community health nursing.

### Major themes and sub-themes

Three themes were identified: (1) attributes of quality nursing care, (2) barriers to quality nursing care, and (3) impact of quality nursing care. Quotes are attributed using a coding system in which the hospital identifier appears first, followed by participant type and number. For example, MPN1 denotes Hospital M, Professional Nurse, Participant 1.

#### Theme 1: Attributes of quality nursing care

Participants defined quality nursing care as holistic, patient-centred and professionally grounded, supported by clinical competence and adequate resources. Nurses emphasised that quality care promotes health, ensures positive outcomes and respects patients’ rights. It is the best care nurses can offer as one participant expressed:

‘Quality nursing care is the best we can offer to improve health outcomes.’ (MPN1, 30 years of age, male, diploma)

Participants expressed key elements of quality care, which included the need for individualised, standard based care, holistic approach, patient rights and involvement, core nursing practices, professional standards and ethics, continuous professional development, resource availability and adequacy, and adequate monitoring and supervision.

**Individualised, standard-based care:** Nurses emphasised the importance of individualised, standards-based care – adapting their approach to each patient’s specific condition by applying clinical expertise and evidence-based best practices:

‘You give the best standard of care based on what you know about the patient’s condition.’ (NM2, female, 24 years of experience)

**A holistic approach:** Quality care was also expressed as a holistic approach, addressing physical, emotional, psychological, social and spiritual needs, with a strong emphasis on privacy, dignity and consent:

‘We make sure we are holistically attending to patients, providing privacy, and listening to their views.’ (SPN2, 28 years of age, male, diploma)

**Patients’ rights and involvement:** Patient rights and involvement through active communication and patient inclusion in decision-making were deemed critical quality care:

‘You involve patients in their treatment and explain every procedure to them.’ (SPN5, 30 years of age, female, bachelor of nursing)

**Core nursing practices:** Core nursing practices in terms of routine but vital tasks such as medication administration, monitoring vitals and wound care were highlighted, alongside concern over negligence and poor attitudes:

‘Some nurses neglect procedures or are distracted by phones. This affects care.’ (RPN6, 29 years of age, male, bachelor of nursing science)

**Professional standards and ethics:** Adherence to the professional standards such as the nursing code of conduct and ethical principles was seen as non-negotiable for maintaining care quality:

‘Every care you render should meet the standard and follow our code of practice.’ (RPN3, 33 years of age, female, diploma)

**Continuous professional development:** Continuous professional development by ongoing education and in-service training were cited as essential to maintaining competence and adapting to evolving care needs:

‘Even short courses help us broaden our knowledge and improve care.’ (MPN2, 27 years of age, female, bachelor in nursing science)

**Resource availability and adequacy:** Access to resources such as basic supplies and functioning equipment was viewed as indispensable to safe and effective care:

‘You can’t deliver quality care without gloves, cannulas, or working equipment.’ (MPN2, 27 years of age, female, bachelor of nursing science)

**Adequate monitoring and supervision:** Participants advocated for stronger monitoring and supervision to uphold standards and correct poor practices:

‘Monitoring and supervision are key – without them, nurses won’t perform as expected.’ (SPN5, 30 years of age, female, bachelor of nursing)

#### Theme 2: Barriers to quality nursing care

Participants identified multiple structural, behavioural and cultural barriers, as well as leadership and accountability gaps that hinder quality nursing care delivery.

### Structural barriers

Structural barriers included inadequate supervision, limited resources, staff shortages and related high workloads, and language challenges.

#### Inadequate supervision

In particular, weak supervision of student nurses was identified as a significant obstacle to delivering quality nursing care, with a noted disconnect between tutors and preceptors and their responsibilities:

‘Students are left unsupervised; there’s a disconnect between tutors and preceptors.’ (NM3, 56 years of age, female)

#### Limited resources

Chronic shortages of essential items disrupt care delivery and burden patients financially:

‘Basic items like gloves or plasters are often unavailable; patients must buy them.’ (MPN5, 32 years of age, female, bachelor of nursing science)

#### Staff shortages and related high workloads

Limited human resources, resulting in high nurse–patient ratios and workload were found as significant barriers to maintaining quality care:

‘We don’t have enough staff to meet patient needs.’ (RPN4, 35 years of age, male, bachelor of nursing science)

‘The poor nurse-patient ratio worsens the burden and reduces the quality of care.’ (NM1)

#### Language challenges

Serving linguistically diverse patient populations without interpreters impedes effective communication, causing a barrier to quality nursing care:

‘None of us could speak the patient’s language; we had to find an interpreter.’ (SPN1, 31 years of age, female, diploma)

### Behavioural and cultural barriers

Behavioural and cultural barriers to quality nursing care included poor staff attitudes and communication, a lack of commitment and professional identity, as well as instances of professional jealousy. These factors were perceived as significant obstacles to delivering effective and compassionate care.

#### Poor staff attitudes and communication

Some nurses displayed poor attitudes and communication, resulting in unprofessional behaviour, including rudeness and a lack of empathy:

‘Some use distasteful language that hurts patients.’ (SPN1, 31 years of age, female, diploma)

#### A lack of commitment and professional identity

A lack of commitment and professional identity were expressed where motivation and pride in the profession were seen as lacking among some staff:

‘Some are in nursing for job security, not passion.’ (MPN1, 30 years of age, male, diploma)

#### Professional jealousy

Professional jealousy such as negative attitudes towards enthusiastic or proactive colleagues were expressed to be a barrier towards quality nursing care:

‘You’re tagged as “too knowing” when you go the extra mile.’ (MPN2, 27 years of age, female, bachelor of nursing science)

### Leadership and accountability gaps

Leadership and accountability gaps including a lack of supervision and oversight, and absence of recognition and motivation were also mentioned to negatively affect quality nursing care.

#### A lack of supervision and oversight

A lack of supervision and oversight included inadequate monitoring enables poor practice to persist without consequences, as expressed by one participant:

‘If nurse managers don’t hold us accountable, things won’t change.’ (SPN6, 32 years of age, female, diploma)

#### Absence of recognition and motivation

Participants felt unappreciated and demoralised by the lack of incentives and acknowledgment, as expressed by one nurse participant:

‘No one sees what you do right – only the mistakes are highlighted.’ (MPN2, 27 years of age, female, bachelor of nursing science)

### Theme 3: Impact of quality nursing care

Participants highlighted the dual impact of nursing care on both patient outcomes and the overall healthcare system. The effects of quality nursing care – whether positive or negative – were predominantly discussed by nurse participants, with comparatively fewer insights offered by nurse managers.

#### Negative impacts of poor care

Participants highlighted several adverse outcomes associated with poor-quality care, including increased hospital readmissions. These challenges not only strain healthcare systems but also place a significant burden on patients and their families, as expressed by the following participant:

‘Patients come for review and end up being readmitted – this increases their burden.’ (MPN4, 28 years of age, male, bachelor of nursing science)

Delays in treatment and inadequate care were seen to worsen patients’ conditions, often resulting in avoidable deterioration and prolonged hospital stays, expressed as follows:

‘Delays in care worsen conditions and extend hospitalisations.’ (RPN6, 29 years of age, male, bachelor of nursing science)

#### Positive impacts of quality care

In contrast, the provision of quality care was associated with a range of positive outcomes. Participants emphasised improvements in patient recovery, satisfaction and overall well-being, as expressed by the following participant:

‘There’s no joy like seeing a critically ill patient walk home healthy.’ (RPN5, 31 years of age, female, diploma)

Quality care was also seen to enhance health literacy, empowering patients to take an active role in their treatment and recovery process. This collaborative approach fosters better adherence to care plans and contributes to improved health outcomes:

‘When we educate patients, they become part of the healing process.’ (MPN4, 28 years of age, male, bachelor of nursing science)

## Discussion

This study explored the perceptions of nurses and nurse managers regarding quality nursing care in public hospitals in the Bono Region of Ghana. The findings indicate that quality nursing care is attributed to holistic and patient-centred care, underpinned by professionalism, clinical competence and ethical conduct. Consistent with previous studies (Fredericks & Naidoo 2023; Lateef & Mhlongo 2022), participants emphasised that quality nursing care involves the delivery of individualised, effective and efficient services that address patients’ physical, emotional and spiritual needs, while promoting patient participation and respect for patient rights. Participants further highlighted the need for adherence to professional codes, standards and ethical frameworks while performing core nursing practices. This supports the findings of Oldland et al. (2020), who reported that ethical compliance strengthens patient trust and enhances the safety and effectiveness of nursing care. In addition, participants highlighted continuous professional development as a key factor contributing to the quality of nursing care. This finding aligns with Shaw (2023), who argues that sound clinical decision-making and improved care outcomes are enhanced when nurses receive continuous training and professional updating. Participants also associated the delivery of quality nursing care with the availability and adequacy of material resources, observing that sufficient equipment enable timely, effective patient care. They further highlighted the importance of adequate monitoring and supervision, where structured oversight, feedback and performance monitoring help to ensure that care standards are upheld, support professional development and contribute to improved clinical outcomes. Research shows that adequate resources are key elements of a positive practice environment and are closely linked to perceptions of care quality, while effective supervision and delegation practices enhance teamwork, accountability and safe nursing care delivery (Rivaz et al. 2017).

Numerous barriers to quality nursing care were identified, particularly structural challenges. These included limited resources, including staff shortages, inadequate medical supplies and limited equipment. Similar challenges have been reported in other studies conducted in Ghana (Yakubu et al. 2022), which can reinforce increased workload, turnover and migration pressures, affecting their ability to provide optimal care. For example, literature on nurse emigration in Ghana indicates that it has led to higher nurse-to-patient ratios, intensifying workload pressures and contributing to emotional exhaustion, diminished morale and overall job dissatisfaction (Alhassan et al. 2025; Coudounaris, Akuffo & Nkulenu 2020). In addition, participants emphasised that inadequate supervision and language challenges affect the quality of nursing care. They advocated for regular in-service training, mentorship programmes and strengthened managerial oversight to enhance accountability and skill development. These findings are supported by Brás Baptista Sérgio, Rodrigues Faria de Carvalho and Correia Barroso Pinto (2023), who demonstrated that effective clinical supervision positively influences key quality indicators in nursing practice. Language barriers can significantly compromise the quality of nursing care by causing miscommunication, incomplete assessments and delays in treatment, ultimately affecting patient safety and satisfaction (Gerchow et al. 2021).

The study also revealed behavioural and cultural challenges within nursing teams as barriers towards quality nursing care. Participants reported negative staff attitudes, poor communication, a lack of professional commitment and identity, and professional jealousy such as interpersonal conflict as factors undermining the provision of quality nursing care. These issues contributed to workplace tension and negatively affected team cohesion and care delivery. This observation is consistent with Meneses-la-Riva et al. (2025), who emphasise the importance of nurse leadership in addressing relational barriers to foster collaborative and supportive practice environments.

Leadership and accountability gaps were identified as significant barriers to quality nursing care, with participants reporting that a lack of effective supervision and oversight undermined clinical performance, hindered professional support and negatively affected care standards. They also found that the absence of recognition and motivational strategies for nurses diminished morale and reduced accountability for high–quality practice, reflecting wider evidence that weak leadership and inadequate supervisory practices compromise staff engagement and the consistent delivery of safe, high–quality care (Alhassan et al. 2025).

The dual impact of quality nursing care on patient outcomes and well-being and satisfaction was clearly articulated. Poor-quality care resulted in adverse patient outcomes, including re-admissions, delays in care, worsened conditions and prolonged hospitalisation, resulting in additional strain on patients and the overall health systems. Conversely, high-quality nursing care was associated with enhanced patient recovery, satisfaction and overall well-being as well as improved health literacy, empowering patients. These findings are supported by Azevedo et al. (2020) and Enweronu-Laryea et al. (2018), who highlighted the direct relationship between nursing care quality and both individual- and system-level health outcomes.

There is a critical need to implement structured in-service training, mentorship programmes and continuing professional education to ensure nurses remain current with evolving evidence-based practices and standards of care. Hospital management, in collaboration with the Ministry of Health, should prioritise the consistent availability of essential medical supplies and functional equipment, which are fundamental to safe and effective care delivery. Strengthening supervisory systems and introducing performance monitoring mechanisms may further enhance accountability and clinical practice standards. In addition, recognising nurses’ contributions, providing opportunities for career advancement and offering psychosocial support are essential for improving morale, retention and workplace satisfaction.

At both practice and policy levels, stronger nursing support systems are required to sustain quality care. In practice, supportive supervision, continuous professional development and positive organisational cultures are vital. Nurse leaders should promote teamwork, uphold ethical standards and ensure adequate resource allocation. At the policy level, addressing staff shortages, workforce migration and infrastructure deficits requires coordinated national strategies. Priorities should include improving nurse-to-patient ratios, implementing retention incentives and investing in essential equipment and supplies. Integrating nurses’ perspectives into decision-making and institutionalising mentorship, supervision and professional development initiatives can strengthen accountability, patient safety and health system performance. In Ghana, such reforms are essential for advancing Sustainable Development Goal 3 on health and well-being.

### Strengths and limitations

This study offers valuable insights as one of the few qualitative inquiries focused specifically on the perceptions of both frontline nurses and nurse managers in the Bono Region of Ghana. By drawing data from multiple public healthcare facilities, the study captures a diverse range of perspectives, providing a more nuanced understanding of the factors influencing quality nursing care. The use of face-to-face interviews further strengthened the research by eliciting rich, in-depth narratives that reflect the complex realities of care delivery in these settings. However, several limitations should be observed. The study was confined to three public hospitals in a single region, which may limit the generalisability of the findings to private, mission-based or healthcare facilities in other regions of Ghana. In addition, participants’ responses may have been shaped by social desirability bias, especially when discussing institutional weaknesses or personal performance. While the qualitative design allowed for depth of exploration, the small sample size restricts the ability to draw broader statistical inferences. Moreover, cultural norms and hierarchical structures within the healthcare system may have discouraged open criticism of management or disclosure of negative experiences.

Therefore, future research should consider expanding the scope of investigation to include a broader range of healthcare institutions, such as private and teaching hospitals, to allow for comparative analysis of perceptions across different facility types. Quantitative follow-up studies are recommended to measure the prevalence and impact of the barriers and facilitators identified in this study, thereby strengthening the generalisability and applicability of findings. Furthermore, incorporating patient perspectives within the same settings would offer a more holistic understanding of care quality and support the development of patient-centred improvement strategies. Intervention-based studies that evaluate the effectiveness of continuous professional development programmes, enhanced supervision frameworks and improved resource allocation could provide practical insights into improving nursing care outcomes. Finally, further exploration of the effects of brain drain and persistent staffing shortages on nurse well-being and patient safety is crucial to inform evidence-based policy interventions at the national level.

## Conclusion

This study offers valuable insights into how nurses and nurse managers in public hospitals in the Bono Region perceive and experience quality nursing care. Their accounts reveal that quality nursing care is a multifaceted concept encompassing not only clinical competence and ethical practice but also organisational support and effective teamwork. Participants further emphasised that quality nursing care depends on effective leadership, adequate resources and supportive supervision, which collectively enhance patient outcomes, satisfaction and well-being; conversely, deficiencies in these areas can compromise care quality and lead to poorer patient outcomes. The findings suggest that improving nursing care delivery in these settings will require more than individual effort – it demands systemic change. Targeted interventions such as enhanced supervision, regular training and improved interprofessional collaboration are essential. Moreover, overcoming structural and cultural barriers will require a coordinated response from hospital administrators, policymakers and frontline healthcare providers. By prioritising adequate resource provision, supportive work environments and continuous professional development, stakeholders can foster a culture of excellence in nursing care that benefits both patients and the health system as a whole.
